# Nuclear Roles of Spliceosome-Associated microRNAs in Neuronal Cancer Cells

**DOI:** 10.3390/ijms26178349

**Published:** 2025-08-28

**Authors:** Shelly Mahlab-Aviv, Keren Or Swissa, Maram Arafat, Keren Zohar, Ayelet Rachel Peretz, Michal Linial, Ruth Sperling

**Affiliations:** 1 Department of Biological Chemistry, The Life Science Institute, The Hebrew University of Jerusalem, Jerusalem 91904, Israel; shelly.mh@gmail.com (S.M.-A.); keren.zohar@mail.huji.ac.il (K.Z.); 2 Department of Genetics, The Life Science Institute, The Hebrew University of Jerusalem, Jerusalem 91904, Israel; kerenor.swissa@mail.huji.ac.il (K.O.S.); maram.arafat@mail.huji.ac.il (M.A.);

**Keywords:** lncRNA, antisense, nuclear miRNA, spliceosomal-miRNA, splicing, pre-miRNA, miRNA biogenesis, miRCancer

## Abstract

MicroRNAs (miRNAs) are well known for regulating translation in the cytoplasm, yet their nuclear roles remain poorly understood. Previously, we identified spliceosome-associated miRNAs implicated in tumorigenesis and metastasis in breast cancer models. Here, we investigate their nuclear functions in the immortalized human cortical neuron (HCN) cell line, along with glioblastoma (U87MG) and neuroblastoma (SH-SY5Y) cell lines, both widely used as models for brain cancer research. Our findings reveal that spliceosome-associated miRNAs mark neuronal cancer cells and uncover novel nuclear targets. Notably, some spliceosomal miRNAs exhibit opposing regulatory effects in the nucleus compared to the cytoplasm, while others demonstrate potential novel nuclear functions. A prominent example is miR-99b, which overlaps the 5′ splice junction of the poorly characterized long non-coding RNA (lncRNA) sperm acrosome-associated 6 antisense RNA1 (SPACA6-AS1) and, through base pairing, enhances SPACA6-AS1 pre-mRNA levels. These results highlight the diverse and context-dependent functions of nuclear miRNAs in gene regulation and cancer progression, broadening our understanding of their regulatory potential beyond the cytoplasm.

## 1. Introduction

MicroRNAs (miRNAs) are small, ~22 nt non-coding RNAs (ncRNAs) that regulate gene expression, and altered expression has been linked to numerous human diseases, including cancer [1,2]. Their canonical role in mammals is translational inhibition, achieved through base pairing with the 3′-UTR of target mRNAs in the cytoplasm [3,4,5,6,7]. In humans, many miRNA genes are located in introns, and their canonical biogenesis from Pol II transcripts involves nuclear cleavage of the pri-miRNA by Drosha, together with DiGeorge Syndrome Critical Region 8 (DGCR8), to generate a 70–80 base precursor (pre-miRNA) stem loop [8,9,10,11,12]. Following this step, the pre-miRNA is exported to the cytoplasm, where Dicer cleaves it into a mature miRNA that is loaded onto the RNA-Induced Silencing Complex (RISC) [3,4,13]. RISC-bound miRNAs then target mRNAs in the cytoplasm [7]. The dysregulation of miRNA expression can perturb cellular pathways, contributing to diseases such as neurodegenerative disorders, chronic inflammation, metabolic conditions, and cancer [1,2,14,15].

The role of miRNAs in cancer progression is well established. Most studies focus on key cancer-related miRNAs that regulate hallmarks such as proliferation, apoptosis, invasion, and differentiation [16]. Their most direct effects in cancer, especially in gliomas and medulloblastomas, involve the regulation of oncogenes and tumor suppressors. For example, miR-21 is often upregulated in glioblastoma multiforme (GBM), where it targets PTEN and PDCD4, driving proliferation and tumor progression [17]. miRNAs also influence invasion and metastasis: miR-10b enhances glioma cell invasiveness by targeting Homeobox D10 (HOXD10) [18]. While abundant miRNAs are frequently overexpressed in cancer due to relaxed epigenetic control, others are suppressed. For instance, downregulation of miR-9 has been linked to increased metastatic potential in medulloblastomas, by enabling the full activity of the metastasis-associated gene E-cadherin. Conversely, miR-34a is often suppressed in GBM, where it inhibits tumor growth by targeting cell cycle and apoptosis regulators (e.g., Notch1 and Bcl-2). Restoring miR-34a expression with mimics reduces tumor growth in preclinical GBM models, underscoring its therapeutic potential [19].

The ability of miRNAs to suppress or promote tumorigenesis depends on factors such as expression levels and maturation. Their abundance and composition are shaped by biogenesis and subcellular localization (e.g., distribution between nucleus and cytosol) [20]. In addition, crosstalk between RNA-binding proteins and miRNAs provides another layer of post-transcriptional regulation in cancer [21].

The discovery of mature miRNAs in the nucleus [22,23,24,25,26] revealed nuclear functions beyond their classical cytoplasmic roles [27,28]. Active shuttling of miRNAs between cytoplasm and nucleus has also been demonstrated [29,30]. Although their nuclear roles remain incompletely defined [26], miRNAs have been implicated in regulating ncRNAs [31,32,33,34,35], transcriptional silencing [36,37], activation [38,39,40,41], and inhibition [37]. Analyses of miRNA–mRNA–AGO (argonaute) interactions further showed extensive AGO–miRNA mapping to intronic sequences [42]. Many miRNA genes are intronic, while others are transcribed from independent Pol II promoters [6,43,44]. For most intronic miRNAs, both mRNA and miRNA can arise from the same transcript. However, when a pri-miRNA lies within an exon or overlaps a splice site, either the mRNA or the miRNA is produced. In cases of alternative splicing (AS), both can be expressed [44]. Several studies have also demonstrated direct links between splicing and miRNA processing [30,45,46,47,48,49].

Splicing and alternative splicing (AS) are central to the regulation of mammalian gene expression, and AS alterations are common in human diseases, including cancer [50,51,52,53]. Splicing occurs in the nucleus within the supraspliceosome, a massive (~21 MDa), highly dynamic molecular machine that governs all nuclear pre-mRNA processing [54,55]. The supraspliceosome consists of four native spliceosomes interconnected by pre-mRNA [56,57]. Each pre-mRNA, regardless of size or intron count, is individually assembled into supraspliceosomes, ensuring coordinated regulation of processing events [54,55]. Importantly, miRNAs have also been detected within the supraspliceosome [58,59,60], where cross-talk between splicing and miRNA processing has been demonstrated [49,58,59,60,61].

Here, we analyzed miRNA sequences present in supraspliceosomes (SF) isolated from neuronal cancer cells using RNA-Seq. The analyses revealed a broad repertoire of spliceosomal miRNAs in each cell line, with both abundance and type distinguishing them. We further identified nuclear-specific roles for SF-miRNAs distinct from their cytoplasmic functions. A striking example is spliceosomal miR-99b, which overlaps the 5′ splice junction of the poorly characterized lncRNA SPACA6-AS1. miR-99b is expressed from the same genomic region of SPACA6-AS1, yet in the opposite direction, portraying potential base pairing. We show here that SF-miR-99b enhances SPACA6-AS1 pre-mRNA levels. These findings highlight the importance of miRNA positioning in transcriptional regulation and point to new nuclear targets in cancer.

## 2. Results

### 2.1. Isolation and Sequencing of Spliceosomal RNA from Neuronal Cell Lines

Hundreds of miRNA sequences have been identified in supraspliceosomes [58,59], but their functions remain unclear. We analyzed neuronal-origin cell lines derived from glioblastoma (U87MG) and neuroblastoma (SH-SY5Y), as well as HCN, a cortical neuron line used as a model for healthy neuronal function. Nuclear supernatants enriched with supraspliceosomes were prepared under native salt conditions and fractionated on glycerol gradients, as previously described [56] (see Section 4). This protocol conserves the higher-order structure of splicing complexes, as demonstrated by electron microscopy [56,62]. Supraspliceosomes position in the gradient was identified, as before, by their sedimentation at 200S together with splicing factors [58,59,63,64,65].

Small RNAs (<200 nt) were then extracted from the SF of each neuronal cell line and used to construct barcoded small RNA libraries for sequencing, as previously described [58,59]. Three independent libraries were generated for biological triplicates of each cell line. A statistical summary of the RNA-seq libraries is provided in Supplementary Table S1. Alignment of SF sequences to the human transcriptome revealed a complex set of RNA species, including pre-miRNAs, small nucleolar RNAs (SNORDs) [66], intronic sequences, and others. Here, we focus only on reads aligned to the hairpin precursor sequences of primary miRNAs, as defined by miRBase [15], referred to in this study as SF-miRNAs.

### 2.2. Changes in Expression of SF-miRNA Sequences in Neuronal Cancer Cells

Sequencing and alignment of all transcriptome and miRNA collections revealed a large complexity of the miRNA sequences. The mapped SF-miRNAs of the three cell lines, HCN, U87MG, and SH-SY5Y, resulted from the raw data that reported on 688 different miRNA sequences. Among these, 455 miRNAs displayed minimal expression levels (Supplementary Table S1). We also removed measurements that were assigned to miR-6087 (see Section 4). The heatmap includes 455 miRNAs with a minimal read size of 17 nt and a minimal expression of ≥30 CPM across the three cell lines, following normalization (Supplementary Figure S1). The number of counts was normalized among cells (see Section 4). The heatmap scaling of the 9 samples is a log10 transformation of the expression counts (Supplementary Figure S1). We concluded that the nuclear miRNA sequences in all three cell lines are substantially different, with a higher diversity of miRNAs in SH-SY5Y relative to U87MG. The most restricted set of miRNAs is associated with HCN.

**Figure 1:**
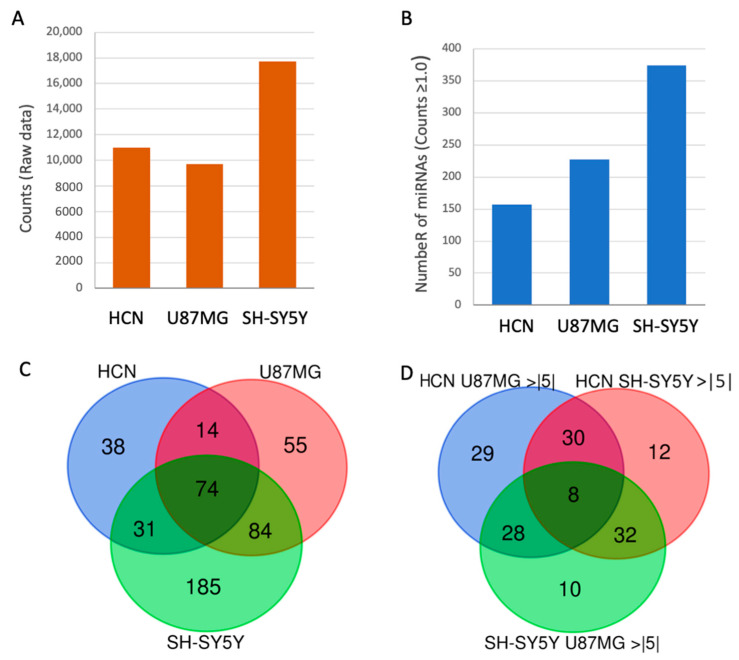
Changes in expression of SF-miRNAs among neuronal cancer—origin cell lines. (**A**) Changes in number of reads. (**B**) Changes in the number of SF-miRNA of the 3 neuronal cell lines (above a minimal expression level). (**C**) Venn diagram showing the partition of different SF-miRNAs among three neuronal cancer cell lines. (**D**) Venn diagram showing the partition of different SF-miRNAs among the three neuronal cancer cell lines showing at least 5× fold change (for up and down regulation).

Figure 1A presents the reads of the raw data of SF-miRNAs observed among the three tested cell lines. Based on the thresholds used for reliably sequenced reads, we identified 155, 227, and 374 SF-miRNAs in HCN, U87MG, and SH-SY5Y cells, respectively (Figure 1B). We found that 74 miRNA sequences were expressed in all cell lines (Figure 1C). The highest expressing miRNAs include miR-1246, miR-21, miR-20a, miR-7704, let-7c, and additional let-7 family members (Supplementary Table S2). After testing the miRNAs that are shared in both HCN and U87MG, an additional 14 miRNAs were found, among them are miR-222, miR-221, and miR-100. An additional 31 SF-miRNAs were shared between HCN and SH-SY5Y (e.g., miR-24-1, miR-24-2, miR-218-1, miR-218-2, miR-27b, and miR-269-2). For the two cancerous cell lines (U87MG and SH-SY5Y), an additional 84 SF-miRNAs were detected (e.g., miR-19b-1, miR-20a, miR-129-2, miR-1291, miR-3198-2, miR-3687). Figure 1C further shows that many of the miRNAs are exclusively expressed in a specific cell line. A total of 38, 55, and 185 SF-miRNAs were expressed solely in HCN, U87MG, and SH-SY5Y, respectively. Notably, among the SF-miRNAs, SH-SY5Y cells exhibit twice as many miRNA types relative to HCN cells (Figure 1B). A Venn diagram of a subset of SF-miRNAs that show a 5-fold change (increase or decrease) is presented in Figure 1D. We concluded that the type and level of expression of SF-miRNAs signify each neuronal cell line and determine the specific miRNA-based nature of the cell.

Figure 1C further shows that many of the miRNAs are exclusively expressed in a specific cell line. A total of 38, 55, and 185 SF-miRNA are expressed solely in HCN, U87MG, and SH-SY5Y, respectively. Notably, among the SF-miRNA, SH-SY5Y cells are exhibiting twice as many miRNA types relative to HCN cells (Figure 1B). A Venn diagram of a subset of SF-miRNA that show 5-fold change (increase or decrease) is presented in Figure 1D. We concluded that the type and level of expression of SF-miRNAs signify each neuronal cancer cell lines and determine the specific miRNA-based nature of the cell line.

### 2.3. Comparison of the Differential Expression of SF-miRNAs in the Neuronal Cell Lines

**Figure 2:**
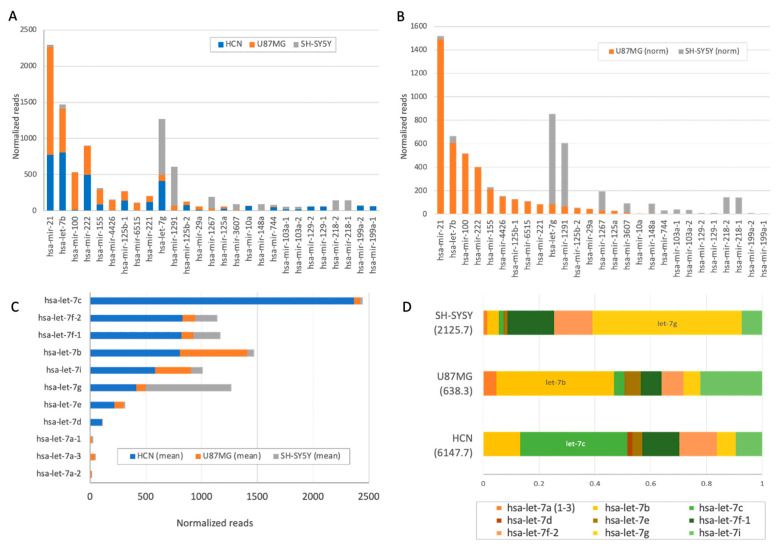
Partition of top SF-miRs in the three neuronal cancer cell lines. (**A**) Changes in the expression level of the top 27 SF-miRNAs in the three neuronal cell lines (log scale). The partitions for each miRNA within the three cell lines are shown. HCN (blue), U87MG (orange) and SH-SY5Y (gray). (**B**) Changes in the expression level of the top 27 SF-miRNAs in the two neuronal cancer cell lines (log scale). Note that the highly expressed miR-1246 was excluded for clarity of the visualization. The partitions for each miRNA within the two cell lines are shown. U87MG (orange) and SH-SY5Y (gray). (**C**) The partitions of SF-miRNAs for let-7 family members within the three cell lines are shown. HCN (blue), U87MG (orange) and SH-SY5Y (gray). (**D**) The relative composition of the different Let-7 SF-miRNA within each of the three neuronal cell lines is presented. Supplemental Table S2 shows the expression level by cell line for the major let-7 family members.

It is known that the average number of miRNA molecules in a cell may vary by 4–5 orders of magnitude [67]. Figure 2 focuses on the difference in expression of the cellular models used, including the HCN and the cancer-originated cells SH-SY5Y and U87MG. As many as 67 SF-miRNAs had a minimal expression level of ≥50 normalized reads (Supplementary Table S1). Figure 2A displays the relative expression levels of 27 instances where SF-miRNA differential expression is substantial for SH-SY5Y and U87MG (fold change > |5|). Figure 2B compares the expression levels of the top SF-miRNAs in the two neuronal cancerous cell lines, demonstrating that the profile of the SF-miRNAs signifies each of these cancerous cell lines.

The analysis further revealed the expression of a large collection of spliceosomal let-7 miRNAs in the neuronal cancer cells. Family members of hsa-let-7 (11 miRNAs) were among the most significantly SF-expressing miRNAs. They accounted for 28% of all reads from the three cell lines. The difference in expression is illustrated in Figure 2C. For example, in the HCN cell line, the total reads for the SF-miRNA let-7 family members were 4.2 times higher than in the other cell lines and accounted for 57% of all expressed miRNAs in this cell. The dominant type of HCN was hsa-let-7c (Figure 2C). The differences in the composition of SF hsa-let-7 in the different neuronal cell lines are evident, with hsa-let-7g, hsa-let-7b, and hsa-let-7c dominating SH-SY5Y, U87MG, and HCN, respectively (Figure 2D).

Table 1 presents the top-listed SF-miRNAs ranked by their normalized expression levels in the three neuronal cells (≥150 reads for each miRNA) along with their functions in cancer of the Central Nervous System (CNS). Among these top-listed SF-miRNAs, over half showed an increased expression in the cancerous versus normal HCN cell lines (e.g., SF-miR-1246, miR-20a, miR-7704). The opposite trend was observed with a third of the SF-miRNAs (e.g., SF-miR-222, miR-320a, miR-221), and only three instances showed increased expression in U87MG cells and decreased expression in SH-SY5Y cells compared to the normal HCN cells (SF-miR-21, miR-100, and miR-155).

We concluded that many miRNAs implicated in tumor-suppressive effects are highly expressed in SF-miRNAs of HCN cells. Furthermore, for about a third of the listed SF-miRNAs, no evidence in CNS tumors has been reported.

### 2.4. Glioma and Glioblastoma Expression Trends

**Figure 3:**
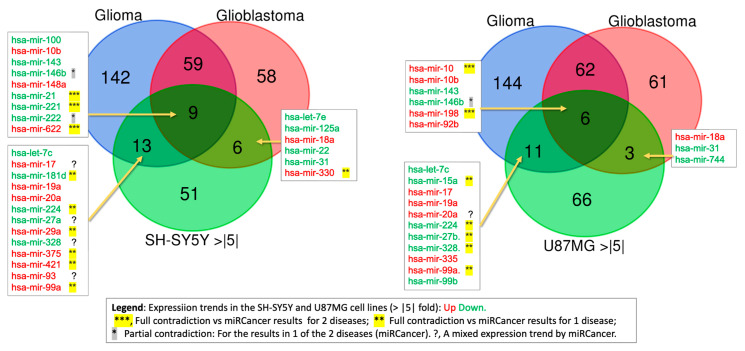
Overlap of miRNA expression trend from CNS cancer and SF-miRNAs from cancerous cell lines. The Venn diagram shows the overlap between differentially expressed SF-miRNAs (≥5-fold change) in SH-SY5Y (**left**) or U87MG (**right**) cell lines, and miRNAs reported in miRCancer database for glioma and glioblastoma. Names of the miRNAs are listed, with the colors marking the expression trend. Up and Down regulation are indicated with red and green font, respectively. The legend indicates the symbols that describes the degree of contradiction of cell line expression trend and the miRCancer data.

We have analyzed the expression trends of miRNAs as reported by miRCancer [68]. The two most common CNS cancers are defined as glioblastoma and glioma (lower-grade glioma, diffuse glioma). These cancer types greatly differ in their aggressive nature and cell origin. While gliomas may originate from astrocytomas, oligodendrogliomas, and ependymomas, glioblastoma (GBM) is a highly invasive type IV astrocytoma with a poor prognosis. We sought coherence between these major human CNS cancers and the differentially expressed miRNAs (DEMs) between the neuronal cancerous cell lines SH-SY5Y and U87MG (Figure 3).

The overlap list is shown in the Venn diagram, with colors indicating the annotation from each cell line (up- or downregulation relative to HCN, in red and green, respectively). For many of our findings, the expression trends reported by miRCancer (compiled from the literature, Supplementary Table S3) contradict the SF-miRNA patterns observed in our cell lines. For example, in SH-SY5Y cells, 9 common SF-miRNAs were identified, among which miR-21, miR-221, and miR-622 displayed opposite directions, while miR-146b and miR-222 agreed with the trend in only one of the two diseases. In addition, 6 SF-miRNAs showed an opposite trend compared with glioma, and one SF-miRNA (miR-330) showed an opposite trend compared with glioblastoma (Figure 3, left). A similar pattern was seen in the U87MG analysis (Figure 3, right). Among the 6 common SF-miRNAs, miR-100 and miR-198 were expressed in the opposite direction to miRCancer, miR-146b matched the trend in only one of the two diseases, and 5 more SF-miRNAs showed the opposite trend relative to glioma.

Although fewer SF-miRNAs matched the glioblastoma trend than the glioma trend in both SH-SY5Y and U87MG (6 and 3 compared to 13 and 11, respectively), the results were generally consistent, with the exception of miR-330, which was upregulated in SH-SY5Y but reported as downregulated in the miRCancer dataset. We also noted that some of the miRCancer entries are themselves conflicting (defined here as >10% of reports showing opposite expression trends). However, even after excluding these conflicted cases (marked by a question mark in Figure 3), more than half still displayed an opposite trend. We conclude that many of the significantly expressed SF-miRNAs in HCN cells likely perform functions distinct from those described in the cytoplasm, and that these roles are linked to their sub-localization within the spliceosome fraction.

Based on the miRCancer analysis, which is based on ample evidence from the literature, we concluded that the subcellular location of miRNAs in the nucleus, specifically at the SF, is a strong indicator of their unique role in the nucleus, irrespective of the classical cytoplasmic impact of miRNAs on the ribosome level and translation.

### 2.5. Changes in the Segmental Regions of SF-miRNA in Neuronal Cancer Cell Lines

**Figure 4:**
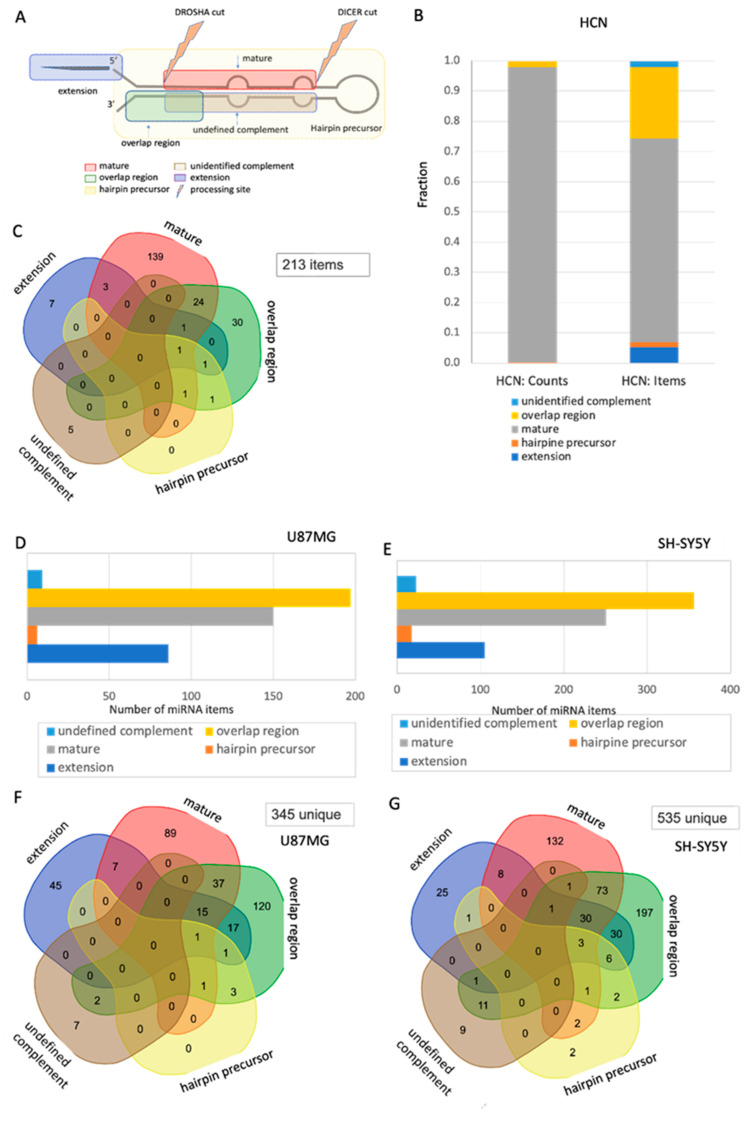
Segmental profile of SF-miRNAs in neuronal cancer cell lines. (**A**) A schematic view of a miRNA prototype, where the different regions of the pre-miRNA are listed according to their positions relative to the pre-miRNA’s major processing sites. An example of an overlap region, defined as reads that cross known segmental border, is indicated. (**B**) The relative counts of reads mapped to each of the predefined regions for the HCN cell line. The relative counts of reads in each of the pre-miRNA regions are color-coded as detailed. Total counts of reads (**left**) and items (**right**) are shown. (**C**) Venn diagram showing the overlap between different segmental groups of SF-miRNA within the HCN cell line. There are 213 unique items. Among the possible 32 sections of the Venn, 20 are marked as zero (i.e., no shared segmental groups). The most significant overlap is associated with the mature and overlap region groups. (**D**) Histogram showing the number of items for the U87MG cell line, categorized by the five types of segment groups. The overlap region is the dominant group. (**E**) Histogram showing the number of items for the SH-SY5Y cell line, categorized by the five types of segment groups. The overlap region is the dominant group. (**F**) Venn diagram showing the overlap between different segmental groups of SF-miRNA within the U87MG cell line. There are 345 unique items. Among the possible 32 sections of the Venn, the most significant overlap is associated with the mature, overlap region, and extension groups. (**G**) Venn diagram showing the overlap between different segmental groups of SF-miRNA within the SH-SY5Y cell line. There are 535 unique items. The most significant overlap is associated with the mature, overlap region, and extension groups. Only 11 of the 32 sections have no shared segments (marked as zero). The data source is found in Supplemental Table S4.

We illustrate the analysis of segmental composition in all tested cell lines. Figure 4A shows the partition of the prototype precursor miRNA into segments (classified into 5 groups). While the 4 segmental groups refer to segments within the pre-miRNA transcript, the extended segment refers to genomic sequences at the margins of the precursor group.

In the case of the HCN cell line, Figure 4B shows that almost all the SF-miRNA-derived sequences are assigned to mature miRNA (98%). By accounting only for the number of unique items (a total of 250 items), the fraction of overlapping regions with other segments is more substantial (33%). Figure 4C provides a detailed analysis of the overlaps between different segmental groups. We observed that the mature miRNAs and overlapping regions exhibit substantial overlap, while the rest of the segmental types are negligible. Altogether, there were 250 references of SF-miRNAs that accounted for 213 unique items (due to having the same miRNA in more than one segmental group). The occurrence of 30 SF-miRNAs that are only identified as overlapping regions and SF-miRNAs that show evidence of unidentified complement sequences (miR-1281, miR-636, miR-4737, miR-555, and miR-3685) highlights alterations in processing and the potential role of nonconventional transcripts at the spliceosome. Unlike SF-miRNA in HCN cells, in which most of the types are mature miRNA, in both neuronal cancer cells (U87MG and SH-SY5Y), the majority of SF-miRNA are overlapping sequences (Figure 4D,E). A detailed analysis of the overlaps among segmental groups in U87MG (Figure 4F) and SH-SY5Y (Figure 4G) shows that the trend in both cancerous neuronal cells is quite identical, with the main segmental regions being overlapping regions, followed by mature sequences. It should be noted that in HCN (Figure 4C), U87MG (Figure 4F), and SH-SY5Y (Figure 4G), we identified among the SF-miRNA 5, 7, and 9 unidentified complement segments, respectively.

### 2.6. Spliceosomal miR-99b Inhibits Splicing of the lncRNA SPACA6-AS1

**Figure 5:**
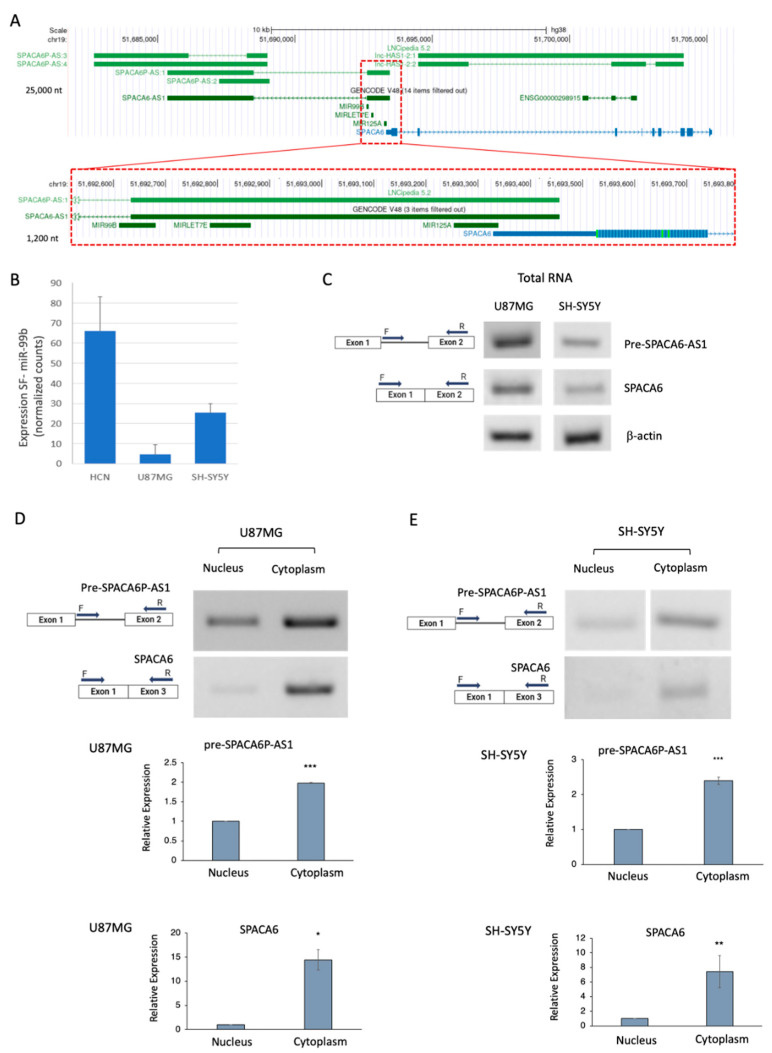
Genomic and gene expression views on SPACA6-AS1 pre-mRNA and the genomic miR-99b in neuronal cancer cells. (**A**) Genome browser of UCSC (hg38) focuses on 22,000 nt from Chr19 covering SPACA6 and SPACA6-AS1. The miR-99b cluster is indicated by the dashed red frame, and zoom-in (red dashed frame) of the 1250 nt around the miR-99b cluster. The ability of miR-99b-5p to completely complement by base pairing to the 5′ splice junction of the intron of SPACA6-AS1, which is transcribed in the antisense direction of SPACA6 is shown. (**B**) RNA-Seq results of SF-miR-99b of the three neuronal cell lines. The differential expression levels across all three cell lines are significant as determined by the adjusted *p*-values (FDR; Supplemental Table S1). (**C**) Results from the RT-PCR analysis of SPACA6 and SPACA6-AS1 pre-mRNA expression from total RNA extracted from the U87MG and SH-SY5Y cell lines are shown. (**D**) RT-PCR analysis of RNA extracted from nuclear and cytoplasmic fractions of the U87MG cell line for the expression of pre-SPACA6-AS1 and SPACA6 (upper panel). Bars represent quantitation of three biological repeats of the respective RT-PCR analyses (lower panel). (**E**) RT-PCR analysis of RNA extracted from nuclear and cytoplasmic fractions of the SH-SY5Y cell line for the expression of pre-SPACA6-AS1 and SPACA6 (upper panel). Boxes, exons; lines introns; arrows mark the position of the PCR primers. Bars represent quantitation of three biological repeats of the respective RT-PCR analyses (lower panel). For statistical significance we labeled ***, ** and * to indicate *p*-values (paired *t*-test) of <0.0001, <0.001, <0.01, respectively.

An interesting case of potential crosstalk through RNA: RNA base pairing between an SF-miRNA and a transcript expressed from the same genomic region, yet in the opposite direction, is the case of miR-99b (Figure 5A). miR-99b is a member of a cluster of miRNAs (miRNA-99b, let-7e, and miRNA-125a), which is antisense to SPACA6-AS1 (Figure 5A). miR-99b is positioned in intron 1 of an isoform of the SPACA6 gene (sperm acrosome-associated 6). Notably, miR-99b fully complements the 5′ splice junction of intron 1 of SPACA6-AS1, which is transcribed in the antisense direction of SPACA6 and miR-99b. SPACA6-AS1 (also termed LINC01129, SPACA6p-AS) is a long non-coding RNA (lncRNA) for which very little is known, and according to GTEx data, its expression is detected in the testis (Supplementary Figure S2). It is considered a risk lncRNA, inversely related to breast cancer survival [69]. SPACA6-AS1 also forms a competing endogenous RNA (ceRNA) network, involving miR-125a and its mRNA targets (Lin-28 Homolog B (Lin28b), Matrix Metallopeptidase 11 (MMP11), Sirtuin 7 (SIRT7), Zinc Finger and BTB Domain Containing 7A (Zbtb7a)) in hepatocarcinoma cells. In these cells, miR-125a can regulate the expression of SPACA6-AS1 and vice versa, and overexpression of SPACA6-AS1 regulates onco-suppressive miR-125a, resulting in the upregulation of its oncogenic targets [70].

Because miR-99b is fully complementary to the 5′ splice junction of intron 1 of SPACA6-AS1, covering positions -6 to +16 relative to the junction and likely forming a 22-nt duplex, this complementarity suggests that SF-miR-99b can inhibit the splicing of SPACA6-AS1 by preventing the binding of U1 and U6 snRNPs, both required for the splicing of SPACA6-AS1 lncRNA. A crosstalk between SF-miR-99b and SPACA6-AS1 has been proposed [58], in which SF-miR-99b might alter the splicing of SPACA6-AS1 pre-mRNA by acting as a splicing inhibitor. While miR-99b targets the 5′ splice junction of SPACA6-AS1, let-7e and miR-125a are complementary to its exon 1.

RNA-Seq of SF-miRNAs in the three neuronal cell lines (Figure 5B) revealed that the expression level of miR-99b is lower in the neuronal cancer cells compared to the normal HCN cells. The expression levels of the other two spliceosomal SF-miRNAs, miR-125 and Let-7e, also decrease in the cancer cell lines compared to the normal HCN cells (Supplementary Table S1). Because each of the three neuronal cell lines is distinct, we focused on each of them separately and tested the effect of miR-99b on each of these two cell lines (the HCN cell line was discontinued and thus was not analyzed for the effect of miR-99b). RT-PCR analysis of total RNA from the U87MG cells revealed the expression of pre-SPACA6-AS1 and SPACA6. Low levels of pre-SPACA6-AS1 and SPACA6 were found in total RNA from SH-SY5Y cells (Figure 5C). We next asked if the pre-SPACA6-AS1 is exported to the cytoplasm in neuronal cancer cells, or if it is retained in the nucleus. For this aim we isolated nuclei and cytoplasm from each of the neuronal cancer cells (see Section 4, Supplementary Figure S3) and analyzed the expression of pre-SPACA6-AS1 and SPACA6 by RT-PCR. Figure 5D,E show that pre-SPACA6-AS1 is found in both the nucleus and cytoplasm in both neuronal cancer cells, but the majority is exported to the cytoplasm.

**Figure 6:**
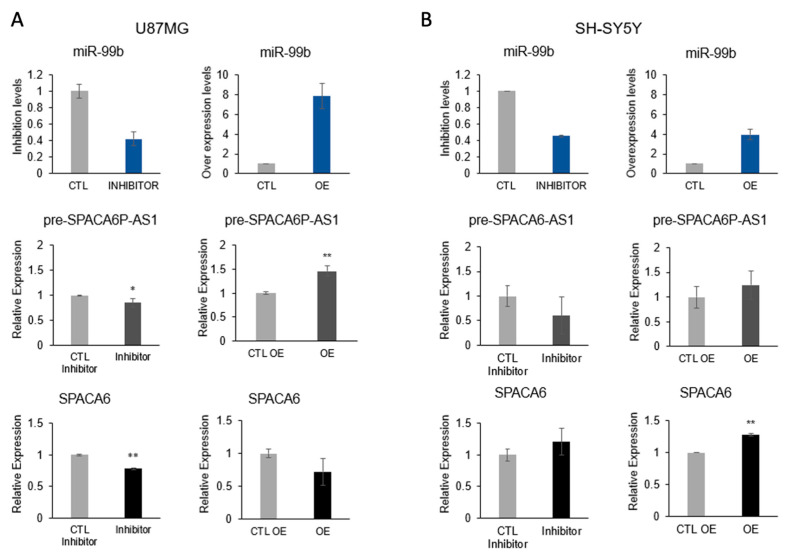
miR-99b increases the level of pre-SPACA6-AS1 in U87mG and SH-SY5Y neuronal cancer cells. U87MG and SHSY5Y cell lines were transfected with anti-hsa-miR-99b inhibitor and pre-miR hsa-miR-99b. The effect of inhibition (**left**), or overexpression (OE, **right**) of miR-99b on the nuclear expression of pre-SPACA6-AS1 and SPACA6 mRNA was analyzed by real time PCR in U87MG (**A**) and SH-SY5Y (**B**) cell lines. The results of quantitative PCR representative of independent biological preparations are shown. The expression levels of SPACA6 and SPACA6-AS1 pre-mRNA was normalized to internal control of ß-actin expression from the same preparation. CTL, control; OE, overexpression; Inhibitor, anti-hsa-miR-99b inhibitor; CTR inhibitor, non-silencing anti-miR (see Section 4); CTR OE, pre-miR negative control (see Section 4). For statistical significance we indicated with ** and * the *p*-values (paired *t*-test) of <5 × 10^−3^, and <5 × 10^−2^, respectively.

To test the effect of miR-99b on the splicing of SPACA6-AS1, we analyzed the effect of inhibition and overexpression of miR-99b on the expression of SPACA6-AS1 pre-mRNA by qPCR in two neuronal cancer cell lines (Figure 6). Attempts to determine also the level of SPACA6-AS1 mRNA failed to produce reliable and stable result (despite design and test of several sets of primers for PCR and qPCR). This might be explained by the extremely low expression level of SPACA6-AS1 in the analyzed cell lines (Supplementary Figure S2). In U87MG cells, inhibition of miR-99b to ~40% resulted in downregulation of pre-SPACA6-AS1, while the effect on the expression of SPACA6 was low. Overexpression of miR-99b increased the expression of pre-SPACA6-AS1 by 50%, while slightly decreasing the expression of SPACA6 mRNA (Figure 6A). In the SH-SY5Y cell line, the similar downregulation of pre-SPACA6-AS1 RNA by the inhibition of miR-99b was observed, and a slight increase in expression of SPACA6. The overexpression of miR-99b in SH-SY5Y cells resulted in increased expression of SPACA6-AS1 pre-mRNA and also an increase in the expression of SPACA6 (Figure 6B). Because miR-99b displays a full complementarity to the 5′-splice-site of SPACA6-AS1, we can conclude that inhibition of miR-99b decreased the level of SPACA6-AS1 pre-mRNA, while overexpression of miR-99b increased the level of SPACA6-AS1 pre-mRNA, likely by affecting the splicing of SPACA6-AS1 pre-mRNA.

The relatively low effect of overexpression of miR-99b on the level of SPACA6-AS1 pre-mRNA in SH-SY5Y cells could be explained either by the complex effect of the cluster of miRNAs (miR-99b, let-7e, and miR-125a) in these cells or by the high percentage of all SPACA6-AS1 transcripts being precursor molecules in SH-SY5Y cells, and thus the effect of overexpression of miR-99b on SPACA6-AS1 pre-mRNA is small. We cannot exclude a combination of both explanations or additional, yet undetermined, causes. These experiments show that the level of SPACA6-AS1 pre-mRNA is directly correlated with the level of SF-miR-99b, indicating that miR-99b, which complements SPACA6-AS1 5′ splice junction, affects its splicing.

## 3. Discussion

The crosstalk between miRNA and the spliceosome was already confirmed by the presence of numerous miRNA sequences within the endogenous spliceosome identified by RNA-Seq [49,58,59,71], and also by the finding of the main microprocessor components, Drosha and DGCR8, within it [49]. We show that not only sequences derived from intronic miRNAs were found in supraspliceosomes, but also a rich collection of sequences derived from miRNAs expressed autonomously, among them many involved in cancer [58]. This finding strengthens the awareness that, in addition to the classical translational suppression role that characterizes cytosolic miRNAs, miRNA sequences within the endogenous spliceosome might have novel roles. We propose a crosstalk through RNA:RNA base pairing between a spliceosomal miRNA and a transcript expressed from the same genomic region, yet in an opposite direction. Support for this hypothesis was provided by the case of miR-7704, whose genomic position overlaps HAGLR, a cancer-related lncRNA. An inverse expression of miR-7704 and HAGLR was shown in breast cancer cell lines [59]. Moreover, inhibition of miR-7704 caused an increase in HAGLR expression in both cervical [58] and breast cancer cell lines [59]. Furthermore, overexpression of miR-7704 decreased HAGLR expression in a cervical cancer cell line [58]. Importantly, several SF-miRNAs from breast cancer cells show an opposite trend to what is known from miRCancer, including the case of SF-miR-7704. Thus, the spliceosomal miR-7704 acts as a tumor-suppressor gene, and the oncogenic lncRNA HAGLR is its nuclear target [59].

In this study, by analyzing spliceosomal miRNAs in the neuronal cell lines, we provide further support for the novel role of SF-miRNAs as nuclear targets in a neuronal context (Figure 1 and Figure 2, and Table 1), where we validate changes between cancer-derived cell lines and normal cells.

Spliceosome-associated miRNAs mark neuronal cancer cells. Each of the neuronal cell lines is signified by the level of expression of SF-miRNA sequences, the types of SF-miRNA sequences, and their pre-miRNA segmental region. There were more types of miRNAs in the neuronal cancer cells compared to the normal HCN cells (155, 227, and 374 SF-miRNAs in HCN, U87MG, and SH-SY5Y, respectively; Figure 1B), with the highest expressing SF-miRNAs including miR-1246, miR-21, miR-20a, miR-7704, let-7c, and additional let-7 family members (Supplementary Table S2). While 74 miRNAs were common to the three neuronal cell lines, some of the SF-miRNAs were specific to only one of the neuronal cell lines (38, 55, and 185 were specific to HCN, U87MG, and SH-SY5Y, respectively; Figure 1). Notably, the let-7 family was prominently represented among SF-miRNAs across all three neuronal cell lines, although each displayed a distinct expression profile. Specifically, SF-let-7c predominated in HCN cells, SF-let-7b in U87MG cells, and SF-let-7g in SH-SY5Y cells (Figure 2).

We compared the expression trend of miRNAs as reported by miRCancer [68] for glioblastoma and glioma, major human CNS cancers, and the differentially expressed miRNAs (DEMs) between the neuronal cancerous cell lines SH-SY5Y and U87MG (Figure 3). The analysis revealed that of the 9 SF-miRNAs common to SH-SY5Y, glioma, and glioblastoma, 3 showed an opposite trend to miRCancer (SF-miR-21, SF-miR-221, SF-miR-622), while 2 (SF-miR-146b, SF-miR-222) showed an opposite trend to only one of the cancers. Furthermore, 6 of the 13 SF-miRNAs common to SH-SY5Y and glioma exhibit an opposite trend with glioma, and a single SF-miRNA also to glioblastoma. The comparison between SF-miRNAs in U87MG cells and miRCancer data shows similar trends, yet with a smaller number of discrepancies, due to the smaller number of SF-miRNAs in this cell line (Figure 1).

Overall, of the 28 SF-miRNAs shared between SH-SY5Y cells and one or both diseases, 3 showed an opposite trend to the two diseases (SF-miR-21, SF-miR-221, SF-miR-622), and 9 SF-miRNAs showed an opposite trend to one of the diseases. In the U87MG cell line, of the 20 SF-miRNAs shared with one or both diseases, 2 (SF-miR-100, SF-miR-198) show an opposite trend to the two diseases, and 6 show an opposite trend to one of the diseases. The discrepancies between SF-miRNAs and miRCancer trends likely arise from several factors. First, our analysis captures spliceosome-associated nuclear miRNAs, whereas miRCancer largely reflects cytoplasmic, bulk-tissue profiles. Second, we should acknowledge the differences in biological context. Cell lines cannot fully capture the heterogeneous nature of patient samples, which may produce divergent patterns. Further, inconsistencies across published reports (as in miRCancer) reflect context dependent variability. Finally, the abundance of instances with an opposite trend might indicate a different nuclear target for the SF-miRNAs than the cytoplasmic ones. Together, these explanations indicate that nuclear miRNAs constitute a distinct functional subset whose roles cannot be inferred solely from cytoplasmic data. This highlights the importance of considering subcellular localization in miRNA studies and suggests that nuclear miRNAs may hold unrecognized regulatory potential in CNS cancers.

In considering different nuclear target for the SF-miRNAs than the cytoplasmic ones, one option for the nuclear target of SF-miRNAs that comes to mind is through complementarity to a genomic sequence not related to the genomic location of the miRNA. An example is SF-miR-320a, which is complementary to the promoter of RNA PolIII D subunit (POLR3D). An inverse correlation between the expression of miR-320a and POLR3D in several cell lines has been shown. Furthermore, transfection of HEK293 cells with miR-320a led to the silencing of POLR3D at the nucleus [36]. In neuronal cells, we found a decrease in the expression of miR-320a in cancer (Table 1).

Search for nuclear miRNAs’ complementarity in human promoters revealed numerous additional targets, suggesting that they may function in transcription inhibition [37]. Another option is through the complementarity of the SF-miRNA to a nuclear transcript (e.g., pre-mRNA), independent of the genomic location of that transcript (see below). An additional option is through full complementarity of the SF-miRNA to a transcript expressed from the same genomic region, yet in the opposite direction, as was shown for SF-miR-7704 and the lncRNA HAGLR [58,59].

The top SF-miRNA in the neuronal cancer cells was miR-1246, which has been shown to have oncogenic roles in a large number of cancers, including glioma (reviewed in ref [72]). It is also upregulated in the neuronal cancer cells, with the highest level in the SH-SY5Y neuroblastoma cell line. In addition to the cytoplasmic reported targets [72], SF-miR-1246 has potential nuclear targets that complement intronic sequences of over 20 pre-mRNAs. One potential target is KAZN (Kazrin, periplakin interacting) pre-mRNA. KAZN mRNA encodes several alternative isoforms, and its protein plays a role in desmosome assembly, cell adhesion, cytoskeletal organization, and epidermal differentiation. Thus, we suggest that SF-miR-1246 might play a role in the balance between the different isoforms. Another potential example is JMY (junction mediating and regulatory protein, p53 cofactor) pre-mRNA. JMY mRNA encodes a protein that interacts with actin and acts as a p53 transcription coactivator. SF-miR-1246, which complements intronic sequences of the JMY pre-mRNA, might affect its splicing. We propose that those SF-miRNAs in each of the neuronal cancer cells tested here, which show an opposite trend to what is known from the literature compiled in miRCancer for glioma and glioblastoma (Table 1 and Figure 3), might be potential candidates for novel nuclear targets that need to be searched for experimentally in the future.

In this study, we inspect SF-miRNA with full complementarity to a transcript expressed from the same genomic region, yet in the opposite direction. The SF-miR-99b is fully complementary to the 5′ splice junction of lncRNA SPACA6-AS1 pre-mRNA (Figure 5A). Very little is known about SPACA6-AS1, which, according to GTEx data, has expression detected in the testis (Supplementary Figure S2). This lncRNA is a risk lncRNA, inversely related to breast cancer survival [69]. Additionally, in hepatocarcinoma cells, SPACA6-AS1 forms a competing endogenous RNA (ceRNA) network with miR-125a and its mRNA targets (Lin28b, MMP11, SIRT7, Zbtb7a), where miR-125a can regulate the expression of SPACA6-AS1 and vice versa. Overexpression of SPACA6-AS1 regulates onco-suppressive miR-125a, resulting in the upregulation of its oncogenic targets [70].

Using qPCR, we show here that in the neuronal cancer cells U87MG and SH-SY5Y, the level of SPACA6-AS-1 pre-mRNA is directly proportional to the level of SF-miR-99b, which complements the 5′ splice junction of SPACA6-AS1 pre-mRNA. Inhibition of miR-99b expression resulted in a decrease in the level of SPACA6-AS1, while overexpression of miR-99b increased the level of SPACA6-AS1 pre-mRNA (Figure 5 and Figure 6). Due to the low expression level of SPACA6-AS1 in the neuronal cell lines (Supplementary Figure S2), further studies in cell systems with more robust expression level of SPACA6-AS1 are required.

Several SF-miRNAs found in ovarian [58], breast cancer [59], and also neuronal cancer cell lines (this study) overlap with lncRNAs expressed from the same genomic region in an opposite direction. These findings suggest a negative regulation of SF-miRNA on the complementary lncRNA target [61]. One example is the case of miR-7704, which negatively regulates the expression of lncRNA HAGLR in breast cancer cells [59]. Another example is SF-miR-99b, which directly affects level of SPACA6-AS1 pre-mRNA in neuronal cancer cells, likely through inhibiting the splicing of SPACA6-AS1 pre-mRNA (Figure 5 and Figure 6). We propose that instances of base pairing of SF-miRNA with complementary RNA sequences, expressed from the same genomic region yet in an opposite direction, present potential novel nuclear targets against cancer yet to be explored.

## 4. Materials and Methods

### 4.1. Cell Lines

Spliceosomes and RNA were isolated from the following cell lines: HCN-2 human cortical neuron cell line (female origin, doubling time 48 h), used as a model for healthy cell function (ATCC (American Type Culture Collection), Manassas, VA, USA, CRL-3592); U87MG, a human glioblastoma cell line (ATCC (American Type Culture Collection), Manassas, VA, USA, HTB-14), one of the most commonly used glioblastoma cell lines (male origin), with a doubling time of approximately 34 h; and SH-SY5Y, a human neuroblastoma cell line derived from a bone marrow biopsy (ATCC (American Type Culture Collection), Manassas, VA, USA, CRL-2266; female origin with a 27 h doubling time), commonly used in cancer research. They exhibit neuronal properties and can be differentiated into more mature neuron-like cells. Cell lines were cultured according to ATCC protocols for cell maintenance.

### 4.2. Isolation of Supraspliceosomes

Supraspliceosomes were prepared, as previously described [56], from nuclear supernatants enriched in supraspliceosomes from the following cell lines: HCN, U87MG, and SH-SY5Y. Briefly, nuclear supernatants were prepared from purified cell nuclei by micro-sonication of the nuclei and precipitation of the chromatin in the presence of an excess of tRNAs. All isolation steps were conducted at 4 °C. The nuclear supernatant was fractionated on 10–45% (*v*/*v*) glycerol gradients. Centrifugations were carried out at 4 °C in a SW41 rotor run at 41 krpm for 90 min [or an equivalent ω^2^t = 2500 (ω is in krpm; t is in h)]. This protocol conserves the intact splicing complexes, as demonstrated by electron microscopy [56,62]. Supraspliceosomes sediment at 200S together with splicing factors [63,64,65]. The gradients were calibrated with the tobacco mosaic virus as a 200S sedimentation marker, used as previously to locate the supraspliceosomes [58,59,63,64,65].

### 4.3. Protein Detection

Western blotting (WB) analyses of nuclear and cytoplasmic fractions were performed as previously described [64]. We used anti-alpha tubulin antibodies AB-7291 (Abcam, Cambridge, UK) visualized with horseradish peroxidase conjugated to affinity-pure Goat anti-Mouse IgG (H+L; Jackson ImmunoResearch Maine, Portland, OR, USA, 1:5000), and anti-H3 histone antibodies (63) visualized with horseradish peroxidase conjugated to affinity-pure Goat anti-Mouse IgG (H+L; Jackson ImmunoResearch, 1:5000) used as previously described [73], and (09-838, Merck, KGaA, Darmstadt, Germany), visualized with horseradish peroxidase conjugated to affinity-pure Goat anti-Rabbit IgG (H+L; Jackson ImmunoResearch, 1:5000).

### 4.4. RNA Isolation from Supraspliceosomes and Deep Sequencing

RNA was extracted as previously described [56] from supraspliceosomes prepared from each of the three different neuronal cell lines: the two neuronal cancer cells, U87MG and SH-SY5Y, and the non-tumorigenic neuronal cell line HCN.

RNA integrity was evaluated using the Agilent (Santa Clara, CA, USA) 2100 Bioanalyzer. Approximately 1 µg of total RNA was used for small RNA library preparation. Following sequential phosphatase and T4 polynucleotide kinase (PNK) treatment, RNA was ethanol-precipitated to enrich for small RNA species. Three independent libraries were constructed using the NEBNext Small RNA Library Prep Set for Illumina (San Diego, CA, USA) (Multiplex Compatible), following the manufacturer’s instructions. Adapters were ligated to both the 5′ and 3′ termini of RNA molecules, after which reverse transcription and PCR amplification were performed to generate sequencing-ready libraries. Amplified products were size-selected on E-Gel^®^ EX 4% Agarose gels (ThermoFisher Scientific (Waltham, MA, USA), #G401004), and fragments smaller than 200 nucleotides were excised and extracted. The resulting libraries were sequenced on the Illumina (San Diego, CA, USA) NextSeq 500 platform. Raw sequencing reads underwent adapter trimming and low-quality read filtering. High-quality sequences were subsequently aligned to miRBase (Release 21). In parallel, filtered fragments were mapped to the human transcriptome (hg19, human reference genome build GRCh37 as represented in UCSC in 2009. The hg19 GTF annotation from UCSC, accessed via Galaxy), which comprises 963,559 exons derived from 45,314 transcripts. Reads mapping to annotated miRNA loci, as defined in miRBase, were retained for downstream analyses.

### 4.5. Next Generation Sequencing (NGS) Analysis

RNA was extracted as described from three independent biological preparations of supraspliceosome fractions derived from each neuronal cell line. Small RNAs (<200 nt) from these samples were sequenced using the standard Illumina NGS protocol. The resulting libraries contained an average of 32, 34, and 47 million reads for HCN, U87MG, and SH-SY5Y cells, respectively. Raw sequencing data were processed by trimming adapters with Cutadapt (v1.13) and filtering low-quality reads using the FASTX toolkit. Reads from the supraspliceosome fractions were then aligned to the human genome (hg19) and to miRBase (v21) using TopHat (v2.1.1), allowing 90% sequence identity and up to two mismatches. Only reads mapping to the start or end positions of annotated miRNA genes were considered. High-quality reads from the three supraspliceosome preparations of each cell line were pooled. From the mapped reads, only sequences ≥ 17 nt in length were retained. miRNA-aligned reads were defined as all high-quality sequences mapping to any pre-miRNA entry in miRBase. We align the reads to human transcriptomes of GENCODE v19/hg19, miRBase (Release 21 June 2014) with 1881 pre-miRNAs and 2588 mature miRNAs. For downstream analyses, only miRNAs with ≥30 mapped reads across all three cell lines were included, ensuring reliable detection given the relatively limited number of supraspliceosome-derived reads. The entry hsa-miR-6087, which has been withdrawn from the miRBase catalog, was excluded from total counts and normalization.

For cell line datasets, mapped read counts were normalized to CPM (counts per million). To mitigate potential biases and data inflation, normalization and differential expression analyses were performed using the DESeq2 package [74]. DESeq2 constructs a statistical model for the observed counts, incorporating a normalization parameter to adjust for library size differences. The DESeq2 coefficients represent group-level differences. Differential expression was evaluated using a likelihood ratio test (LRT), and statistical significance (*p*-value) was determined and adjuster by false discovery rate (FDR). Only miRNAs meeting thresholds for significant differential expression were further analyzed.

The processed dataset has been deposited in ArrayExpress under accession number E-MTAB-15383.

### 4.6. Validation of Gene Expression of SPACA6 and SPACA6-AS1 Pre-mRNA

#### 4.6.1. RT-PCR

RT-PCR was performed on RNA extracted from the cell lines described above, and from nuclear supernatants of the above cells as described [65]. The following sets of primers for SPACA 6 NM_001316994.2 were used: Forward: 5′-GGGGAGAGGATGGAGAGCG-3′ and Reverse 5′-TCATTTTCTCCGCAGCATC-3′, with Tm 62 °C for 35 cycles. The primers for pre-SPACA6-AS1 were: Forward, 5′-GGGCTCAAAGGTGAATCAGA-3′ and Reverse, 5′AGGGCCTTAGTGGAGGTCAT-3′, and run at Tm 58 °C for 35 cycles. The identity of all PCR products was confirmed by sequencing. Each experiment was repeated at least 3 times. The relative abundance was quantified in view of the intensity of the ß-actin that was used as a control. The ß-actin Forward and Reverse primers, for an amplicon of 120 nt, are: 5′-CTGGAACGGTGAAGGTGACA-3′ and 5′-AAGGGACTTCCTGTAACAATGCA-3′, respectively.

#### 4.6.2. RNA Isolation from Nuclear and Cytoplasmic Fractions

For U87MG cells, nuclear RNA isolation was performed as previously described [49]. Briefly, 24 h post-transfection, six-well plates were washed with PBS, followed by the addition of 175 μL of cold RLN buffer (50 mM Tris, pH 8; 140 mM NaCl; 1.5 mM MgCl_2_; 0.5% NP40). Cells were then scraped and transferred to an Eppendorf tube on ice for 5 min. Samples were centrifuged for 2 min at 750 g at 4 °C. The supernatant (cytoplasmic fraction) was collected into a new tube, and the nuclear pellet was subjected to a second centrifugation. For SH-SY5Y cells, a modification of the above protocol was applied. Briefly, SH-SY5Y cells were seeded in 6-well plates, washed with PBS once, followed by the addition of 175 µL of cold RLN buffer with a lower NP40 concentration per well (0.2% instead of 0.5% NP40). The cells were then scraped and transferred to an Eppendorf tube to incubate on ice for 5 min. Centrifugation for 3 min at 750 g and 4 °C was conducted, followed by separation of the nucleus and cytoplasm. The supernatant fraction (cytoplasm) was transferred to new tubes. Re-centrifugation of the nucleus and cytoplasm tubes was performed for better separation.

RNA was then extracted using the Universal RNA Purification Kit (cat #E3598, EURx Gdańsk, Poland) for RT-PCR experiments. For qPCR experiments, we used the miRNeasy mini kit (cat #217004, Qiagen, Hilden, Germany), following the manufacturer’s instructions. All experiments were performed with at least three biological replicates.

### 4.7. Quantitative PCR

#### 4.7.1. Transfection

U87MG and SH-SY5Y cells were each grown in six-well plates. For the downregulation of hsa-miR-99b, the cells were transfected with anti-hsa-miR-99b inhibitor AM11021 (ThermoFisher Scientific (Waltham, MA, USA)) according to the manufacturer’s instructions at 100 nM for 24 h. As controls, we used the same cells transfected with the anti-miR inhibitor negative control #1 (AB-AM17000, ThermoFisher) and non-treated cells. For the overexpression of miR-99b, we used pre-miR hsa-miR-99b, PM11021 (AB-AM17100, ThermoFisher), and as a negative control, pre-miR negative control (AB-AM17110). Transfections were performed using Lipofectamine 2000 reagent (ThermoFisher).

#### 4.7.2. TaqMan microRNA Assay

For RT of miR-99b, the TaqMan MicroRNA Reverse Transcription Kit (AB-43666596, ThermoFisher) was used according to the manufacturer’s instructions. For PCR, we used hsa-miR-99b Primers (TaqMan^®^ MicroRNA Assays INV, SM/PC hsa-miR-99b:000436, AB-4427975) and MultiScribe™ Reverse Transcriptase (ThermoFisher).

#### 4.7.3. RT of mRNA

RT of nuclear RNA was performed using the High-Capacity cDNA Reverse Transcription Kit (ThermoFisher) according to the manufacturer’s instructions, using RT random primers and MultiScribe™ Reverse Transcriptase.

#### 4.7.4. Quantitative PCR Reaction

mRNA and miR-99b levels were measured using the TaqMan Fast Advanced Mix (ThermoFisher) and the following TaqMan Assays with FAM/MGB-NFQ primers/probe: TaqMan MicroRNA Assays INV, SM/PC, hsa-miR-99b_000436 (AB-4427975, ThermoFisher); TaqMan Gene Expression Assays MTO, XS/PC: beta actin: Hs99999903_m1 (AB-4453320); Custom TaqMan Gene Ex Assays, SM/EA, SPACA6-AS1 intron-exon (AB-4331348, ThermoFisher). Primer Fw: 5′- GCCCCCACCAGCTTTAGTATCT-3′, Rev: 5′-GTCTGCGGCTGGCTCTGT-3′, FAM Probe: 5′-GGAGCTCACAGTCTGA-3′. TaqMan Gene Expression for SPACA6: Custom TaqMan Copy Number Assays, SM/PC: ID APXGYD (AB-4400294, ThermoFisher). Assays were performed the manufacturer’s instructions. Amplification was carried out using a QuantStudio 3 Real-Time PCR System for 40 cycles at an annealing temperature of 60 °C. Analysis was performed using the delta-delta C_T_, 2^−ΔΔC^^T^ method. All experiments were performed with at least three biological repeats.

### 4.8. Statistical and Analytical Analyses

Comparisons between two groups were performed using a two-tailed Student’s *t*-test, and comparisons among three or more groups were assessed by one-way ANOVA with Tukey’s post hoc test. All experiments included three independent biological replicates (n = 3). Data are presented as mean ± standard deviation (SD), with error bars indicating SD, and significance was set at α = 0.05. 

For the analytical methods, we created Venn diagrams based on the online tool (https://bioinformatics.psb.ugent.be/webtools/Venn/, 1 August 2025). The heatmaps were produced by a standard visualization R-library (gplots; heatmap.2). DEGSeq statistics used was released in 2023 (version 1.62).

## Figures and Tables

**Table 1:** Nuclear SF-miRNAs with total normalized reads ≥ 150.

SF-miRNA	HCN (%)	U87MG (%)	SH-SY5Y (%)	Total Counts	Function in CNS Cancer ^a^
hsa-mir-1246	3.27	24.73	**72.00**	5963.50	OncomiR: exosome, regulates Wnt/β-catenin and TP53
hsa-mir-21	33.77	**64.92**	1.32	2294.20	OncomiR: cell proliferation, invasion
hsa-mir-222	**55.32**	44.66	0.02	898.40	cell cycle progression
hsa-mir-20a	0.77	**59.41**	39.81	816.70	miR-17-92 cluster: oncogenic: proliferation, invasion,
hsa-mir-7704	5.84	**72.12**	22.04	792.60	U.D., regulating HAGLR
hsa-mir-19b-1	0.25	**60.57**	39.18	788.50	miR-17-92 cluster: oncogenic: proliferation, invasion,
hsa-mir-1291	0.28	11.01	**88.72**	610.60	U.D.
hsa-mir-3198-2	0.00	**54.17**	45.83	593.90	U.D.
hsa-mir-100	2.59	**97.34**	0.09	529.90	tumor suppressor, targeting mTOR, FGFR3, and IGF1R.
hsa-mir-3687	0.87	41.31	**57.84**	423.60	U.D.
hsa-mir-92a-1	13.35	**51.31**	35.36	417.10	miR-17-92 cluster: oncogenic: proliferation, invasion,
hsa-mir-92a-2	14.68	**50.37**	34.92	374.60	miR-17-92 cluster: oncogenic: proliferation, invasion,
hsa-mir-320a	47.03	30.14	22.83	360.00	tumor suppressor: inhibit angiogenesis and migration.
hsa-mir-155	26.82	**67.58**	5.60	314.30	immune response, STAT3 signaling
hsa-mir-27b	**78.53**	5.81	15.69	302.70	conflict: anti-migration. apoptosis, stemness
hsa-mir-125b-1	**52.31**	45.70	1.99	270.90	inhibiting proliferation and regulate apoptosis
hsa-mir-221	**58.17**	41.83	0.00	206.30	driving proliferation, survival, invasion, apoptosis
hsa-mir-3064	7.24	41.43	**51.33**	203.00	U.D.
hsa-mir-1267	0.00	16.05	**83.95**	195.00	U.D.
hsa-mir-423	29.02	37.62	33.37	193.00	cell growth via p21 and p53-related pathways
hsa-mir-24-2	**81.85**	5.85	12.30	182.90	tumor-suppressive effects
hsa-mir-26a-1	**73.28**	11.12	15.67	158.30	tumor-suppressive effects
hsa-mir-26a-2	**71.48**	12.16	16.36	157.10	tumor-suppressive effects
hsa-mir-4426	0.00	**95.90**	4.10	153.80	U.D.

^a^ Function reported from CNS cancer, when available; U.D, undetermined, unknown function or under-studied. In bold face, the cell line that dominates the signal of this SF-miRNA with >50% of the reads.

## Data Availability

Supporting reported results can be found in the Supplementary Tables S1–S4, and Figures S1–S3. The analyzed data were added to ArrayExpress and the data accession number is E-MTAB-15383. URL: https://www.ebi.ac.uk/biostudies/arrayexpress/studies/E-MTAB-15383.

## Supplementary Materials

The following supporting information can be downloaded at https://www.mdpi.com/article/10.3390/ijms26178349/s1.

## Abbreviations

The following abbreviations are used in this manuscript:

CNS	Central nervous system
SNORD	Small nucleolar RNA
FDR	False discover rate
GBM	Glioblastoma multiforme
HP	Hairpin precursor miRNA
lncRNA	Long non-coding RNA
PCR	False discover rate
RISC	RNA-induced silencing complex
RT	Reverse transcription
SF	Spliceosomal fraction
WB	Western blot
