# Supplementary material for: Nuclear Roles of Spliceosome-Associated microRNAs in Neuronal Cancer Cells
Source: Int J Mol Sci. 2025 Aug 28;26(17):8349. doi: 10.3390/ijms26178349 (PMC12428214; doi:10.3390/ijms26178349)
Supplement: Supplementary file 1 [file ijms-26-08349-s001.zip › ijms-3831010-supplementary.pdf]

International Journal of *Molecular Sciences*

MDPI

Supplementary Figures

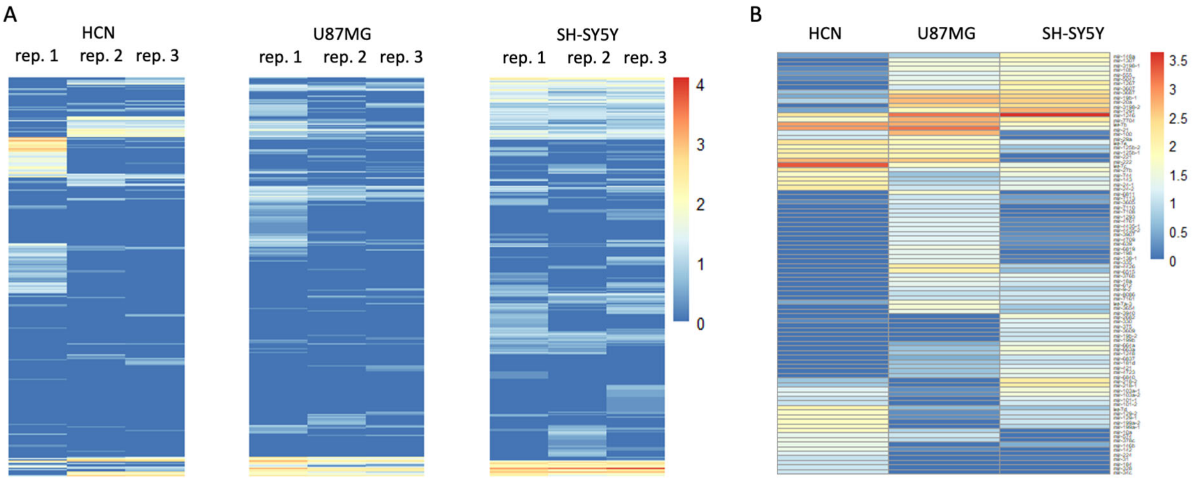

Figure S1. Heatmap of the SF-miRNA of the 3 neuronal cell lines. **(A)** Heatmap of the SF-miRNA of each of the 3 neuronal cell lines, as that met the expression threshold (>30 CPM; total 480 miRNAs). Showed in log10 expression CPM). **(B)** The top 92 miRNAs in which the expression level ratio of CPM+1 is >|10| relative to HCN as a baseline. Data is colored following transformation to log10 (expression ratio). There are 92 miRNAs from miRNAs displayed in A. The data source is in Supplemental **Table S1**.

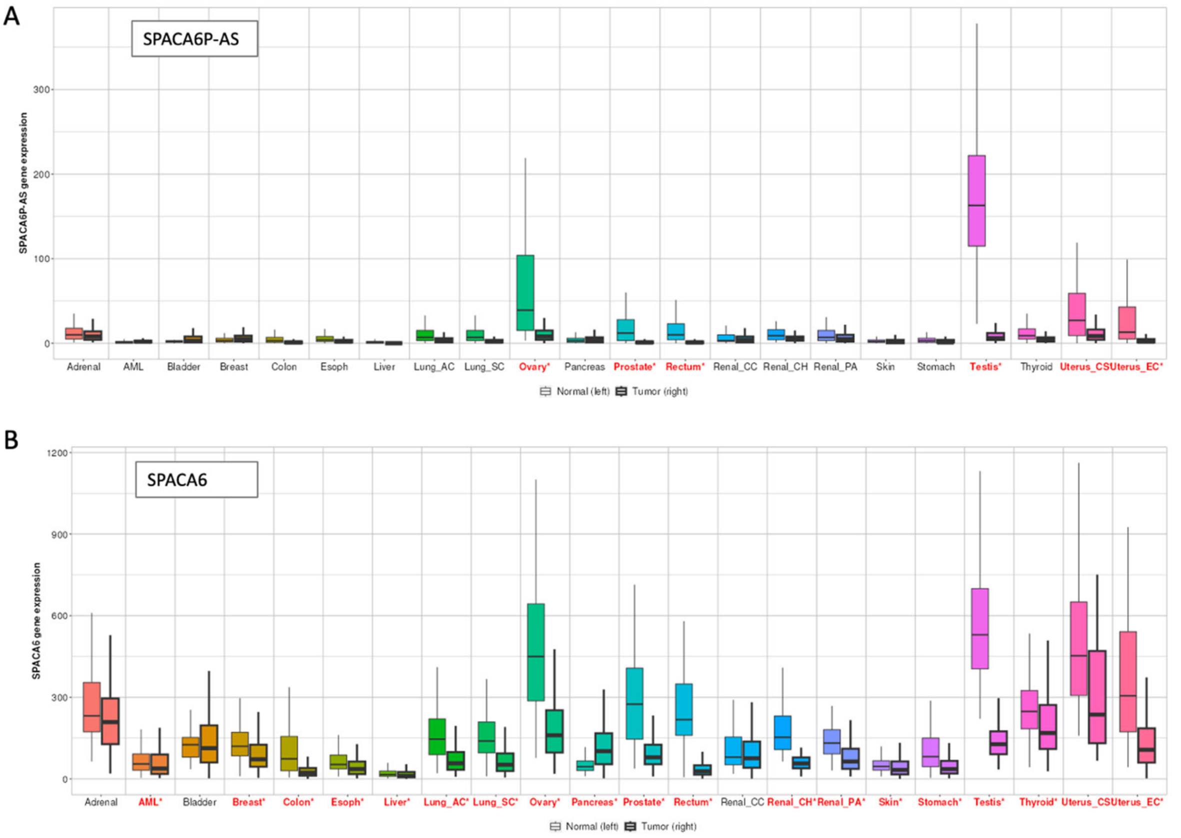

Figure S2. Expression profile of body map of GTEx. The extremely restricted expression level of SPACA6-AS1 across all tissues is shown. Maximal expression is associated with testis. Data is available in https://gtexportal.org/home.

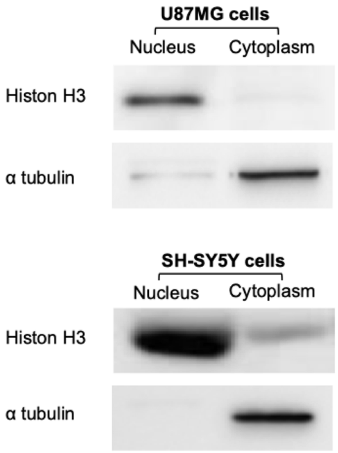

Figure S3. Isolation of nuclear and cytoplasmic fractions from U87MG and SH-SY5Y cell-lines. WB analysis of nuclear and cytoplasmic fractions of U87MG (upper panel) and SH-SY5Y (lower panel) monitoring cell fractionation for nuclear and cytoplasmic fractions.
